# Interpretable machine learning for cattle breed classification and SNP prioritization

**DOI:** 10.1186/s12711-026-01056-7

**Published:** 2026-06-03

**Authors:** Farzad Atrian-Afiani, Gábor Mészáros, Johann Sölkner, Patrik Waldmann

**Affiliations:** 1 Universität für Bodenkultur Wien, Vienna, Austria; 2 Research Unit of Mathematical Sciences, University of Oulu, P.O. Box 8000, 90014 Oulu, Finland

## Abstract

**Background:**

The conservation of endangered cattle breeds is an important priority for maintaining biodiversity and keeping unique genetic resources. Traditional conservation methods are often not precise enough for accurate classification into closely related breeds. The aim of this study was to develop a machine learning classification model using single nucleotide polymorphisms to improve breed identification and to identify breed discriminating markers.

**Results:**

We applied a tuned Light Gradient Boosting Machine (LightGBM) and a Random Forest (RF) classifier to genome-wide SNP data from 6850 individuals representing 11 endangered Austrian cattle breeds. To interpret the model predictions, SHapley Additive exPlanations (SHAP) values were used. Hyperparameters were tuned within the training sets using five-fold cross-validation. Final models were then evaluated on the independent test sets (20% of the data) across six random seeds, yielding mean classification accuracies of 0.842 for LightGBM and 0.837 for Random Forest. Feature importance analysis identified the top 100 single nucleotide polymorphisms (SNPs) contributing most to breed separation. Some SNPs were highly specific for individual breeds, while others reflected broader population genetic structures. In particular, ARS-BFGL-NGS-97995 distinguished major breed clusters, whereas DIAS-308, ARS-BFGL-NGS-843, and ARS-BFGL-NGS-3513 were highly breed-specific for Fleckvieh, Brown Swiss, and Original Braunvieh, respectively.

**Conclusions:**

The machine learning methods provide scalable tools for accurate breed prediction based on genomic data, and the identified SNPs provide practical markers that can support breed management, monitoring programs, and policy strategies. Hence, by combining efficient classification with interpretable feature analysis, our study offers a useful framework for genomic conservation.

**Supplementary Information:**

The online version contains supplementary material available at 10.1186/s12711-026-01056-7.

## Background

Livestock biodiversity is a critical yet often overlooked component of global agricultural heritage. Cattle breeds serve as vital reservoirs of genetic diversity, cultural identity, and economic resilience. However, many indigenous and locally adapted breeds face *genetic erosion*, a decline in genetic diversity driven by inbreeding, crossbreeding with commercial breeds, and shrinking population sizes [1, 2]. This erosion threatens not only the survival of unique breeds but also the loss of adaptive traits such as disease resistance, climate resilience, and suitability for traditional farming systems [1, 3]. Preserving this diversity is essential for the long-term sustainability of the cattle industry, as it supports ecological balance and provides genetic resources for future breeding programs, particularly under changing climatic conditions. Thus, the conservation of endangered breeds is not merely a cultural or ecological concern but a strategic imperative for agricultural resilience.

Accurate breed assignment is a fundamental prerequisite for managing endangered populations, especially when pedigree records are unavailable, which might be a common issue for these breeds [4]. Accurate breed classification supports conservation by identifying individuals for targeted breeding programs aimed at population recovery. The foundational step in genomic breed classification involves identifying breed-discriminative single nucleotide polymorphisms (SNPs), a process currently lacking a consensus methodology. Existing SNP selection strategies include statistical approaches like fixation index (F_ST_) analysis [5], principal component analysis (PCA) [6], and absolute allele frequency differences (δ) [7], alongside machine learning techniques such as Random Forest (RF) algorithms [8]. Following SNP selection, machine learning models, including Support Vector Machines (SVM), K-Nearest Neighbors (KNN), and Artificial Neural Networks (ANN), are commonly employed for breed assignment, often achieving accuracies exceeding 95% [9]. However, selecting informative markers and choosing an appropriate number of SNPs remains challenging. An alternative method based on the genomic relationship matrix avoids explicit SNP selection by using all available markers for breed assignment and can achieve high classification accuracy with low computational demands [10]. Nevertheless, many studies use a subset of markers to enhance model robustness. A key difficulty in this context is the high dimensionality of SNP data, where the number of markers is often much larger than the number of samples. This imbalance can reduce the efficiency of classification methods, as not all markers are necessary to build an accurate model. Moreover, the number of samples to be classified is typically small, which increases the risk of overfitting. Overfitting occurs when a classifier fits the training data too closely to the true training data and therefore performs poorly on unseen samples. Consequently, for high-density SNP panels, using all available markers is often not practical [10, 11]. Many SNP selection methods, such as or PCA, do not always capture complex, non-linear interactions within and between genetic markers. This may explain why some studies have found different sets of informative SNPs when using these methods compared with ML methods such as RF [12]. Advanced methods, like RF and neural networks, have been suggested to improve SNP selection, but there is still no agreement on the best number of SNPs or the optimal selection strategy. In addition, some selected SNPs may not be available on newer genotyping platforms, which raises concerns about reproducibility and long-term use [13]. For these reasons, selecting SNPs requires balancing dimensionality reduction, predictive performance, and future applicability. Tree-based machine learning models efficiently handle high-dimensional data, maintain high predictive accuracy, and are applied in SNP selection and breed assignment. Light Gradient Boosting Machine (LightGBM) is a fast and efficient implementation of the Gradient Boosting Decision Tree (GBDT) algorithm, specifically developed for high-dimensional data and large datasets. Compared with conventional GBDT approaches, which require scanning all data instances to evaluate every possible split for each feature, LightGBM introduces algorithmic optimizations that significantly improve scalability. These improvements allow LightGBM to maintain high predictive accuracy while substantially reducing computational complexity. As a result, LightGBM has become increasingly popular for applications involving large-scale and high-dimensional data, including genomic studies [14]. To make machine learning models more interpretable, SHapley Additive exPlanations (SHAP) provide a game-theory-based method to measure how much each feature contributes to a prediction [15]. By considering all possible feature combinations, SHAP values give consistent and locally accurate explanations, showing which features increase or decrease predictions providing a global understanding of feature importance. Recent developments have made SHAP efficient for complex tree-based models [16], and it has been successfully applied in fields such as medical research and population genetics [17, 18]. In this study, we classify livestock breeds using LightGBM and RF models while skipping the usual initial step of selecting informative SNPs with statistical or population-genetic methods. Instead, we use SHAP values from models trained on all SNPs to identify the most informative markers. We also evaluate predictive performance by testing accuracy with different subsets of informative SNPs obtained from these models, allowing comparison with results using the full SNP dataset.

## Methods

### Breed populations and sampling

The study included 6984 individuals from 11 Austrian cattle breeds, representing a diverse panel of both commercial and locally conserved populations. A summary of breed name and sample size (n), is provided in Table 1. Biological samples were collected following standard animal ethics protocols. Tissue or whole blood samples were obtained by certified veterinarians or local breeding organizations. Genomic DNA was extracted using a standard phenol-chloroform protocol or commercial DNA isolation kits, depending on the sampling site.

**Table 1:** Number of males and females in each breed

Breed	Male	Female	Total
Grauvieh	533	914	1447
Original Pinzgauer	795	591	1386
Pustertaler Sprintzen	360	522	882
Murbodner	502	298	800
Brown Swiss	0	500	500
Fleckvieh	0	500	500
Original Braunvieh	171	279	450
Tuxer	183	169	352
Ennstaler Bergschecken	149	156	305
Kaentner Blondvieh	171	68	239
Waldviertler Blondvieh	91	32	123
Total	2955	4029	6984

### Genotyping and quality control

All animals were genotyped using the Illumina BovineSNP50 BeadChip, which produced an initial dataset of 53,218 autosomal markers. A quality control (QC) pipeline was subsequently applied using PLINK v1.9, where individuals with call rates < 90% (--mind 0.1) and SNPs with call rates < 90% (--geno 0.1), a minor allele frequency < 0.05 (--maf 0.05), or significant deviation from Hardy-Weinberg equilibrium (*p* < 1 × 10^−6^; --hwe 1e−6) were removed. Following QC, remaining missing genotypes were phased and imputed separately within each breed using Beagle v4.6 [19]. This within-breed imputation strategy preserves breed-specific haplotype structure but may also introduce slightly optimistic classification accuracy, as imputation was performed prior to splitting the data into training-validation and test sets. The final dataset consisted of 47,418 high-quality autosomal SNPs from 6850 animals used for downstream analyses.

### Data partitioning

The full genomic dataset was randomly divided into training-validation (80%, *n* = 5482) and independent test (20%, *n* = 1368) subsets. To assess the stability of the results with respect to data partitioning, six independent training-validation and test splits were generated using random seeds ranging from 1 to 6. These training-validation subsets were then used for model development, hyperparameter optimization, and subsequent evaluation on the independent test sets.

### Model development

As a baseline approach, we first applied the GLMNET framework, which fits generalized linear models using penalized maximum likelihood and supports elastic net regularization. GLMNET computes regularization paths for the penalty parameter λ on a logarithmic scale and uses an efficient algorithm that can exploit sparsity in the input data. All analyses were performed in R version 4.5.1 [20]. The GLMNET model served as a reference benchmark for comparison with the more complex ML algorithms, including RF and LightGBM. LightGBM was applied as a tree-based gradient boosting algorithm for model training and SNP importance estimation. It improves the efficiency of standard Gradient Boosting Decision Trees through two key optimizations: Gradient-based One-Side Sampling (GOSS), which prioritizes data instances with large gradients that contribute most to information gain, and Exclusive Feature Bundling (EFB), which reduces the effective number of features by grouping mutually exclusive ones. These strategies allow efficient learning from high-dimensional SNP data while maintaining predictive performance [14].

### Model training and validation

**Fig. 1:**
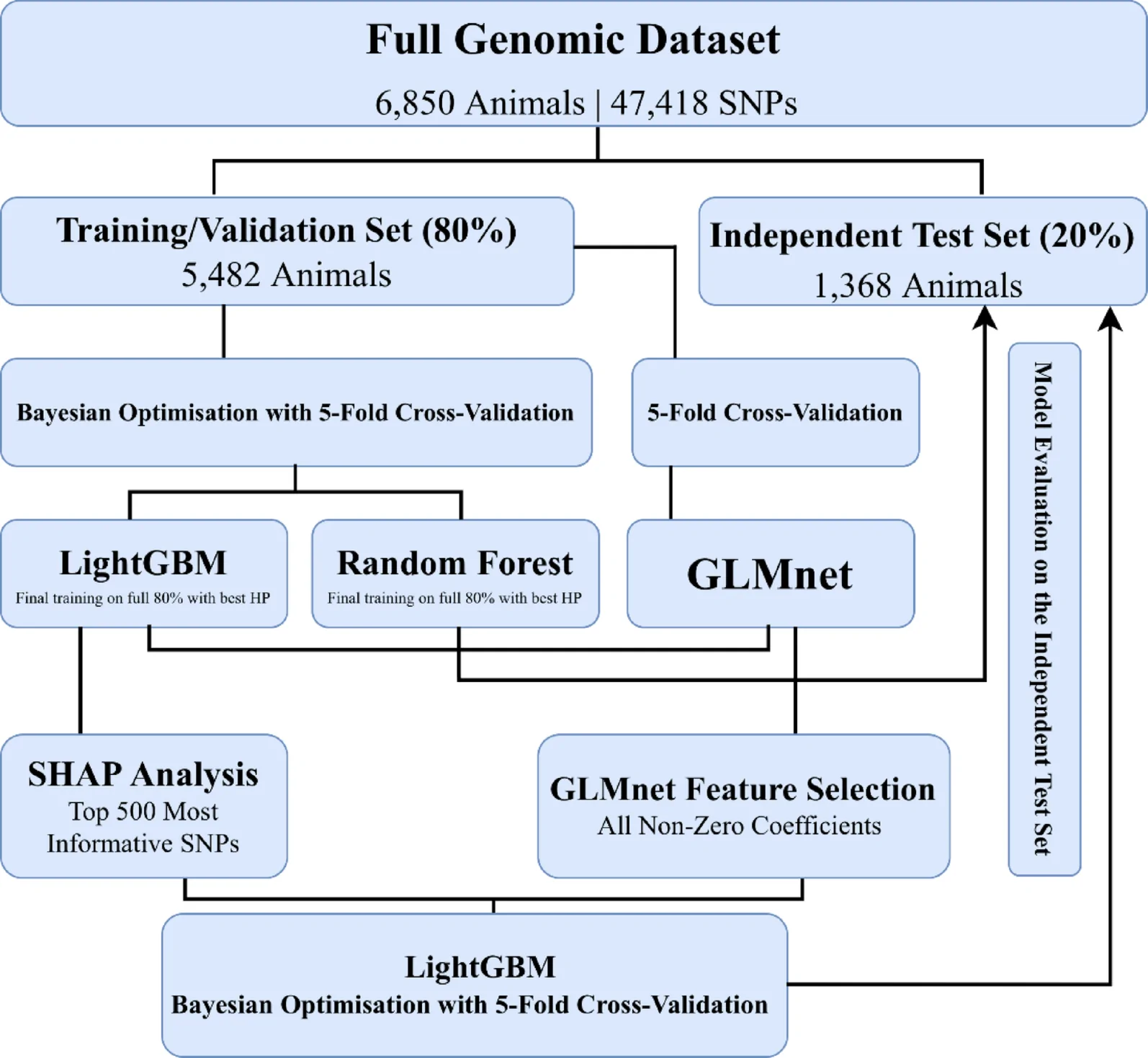
Workflow of data splitting, Bayesian tuning with cross-validation, SNP panel selection (SHAP and GLMnet), and model evaluation on the test set

All model development and hyperparameter tuning were performed using the training-validation partitions of the data. For the GLMNET framework, the regularization parameter (λ) was selected using 5-fold cross-validation implemented through the cv.glmnet function, which evaluates model performance across a sequence of λ values and identifies the value that minimizes the cross-validated multinomial deviance. For both RF and LightGBM, hyperparameters were optimized using Bayesian optimization (BO) combined with repeated 5-fold cross-validation, where the objective function was maximizing validation accuracy for RF and minimizing multi-class log loss (multi_logloss) for LightGBM. BO iteratively builds a probabilistic surrogate model of the relationship between hyperparameters and validation performance and uses this model to guide the selection of new parameter configurations, balancing exploration of uncertain regions with exploitation of promising ones [21]. For RF, the search space included mtry (8000–14,000), min.node.size (1–10), and sample.fraction (0.7–1.0). For LightGBM, optimisation covered the number of leaves (2–25), learning rate (0.001–0.1), and L1 regularisation strength (0.01–0.1). Final training for both models was performed using the optimal hyperparameters obtained from BO. For RF, a final model with 1000 trees was trained on the full training dataset and evaluated once on the independent test sets to obtain test accuracy. For LightGBM, five-fold cross-validation was conducted on the training data to determine the optimal number of boosting iterations with early stopping. The five fold-specific models were then used to generate predictions on the independent test sets, and class probabilities were averaged across folds before calculating test accuracy. The full workflow described above, from data partitioning and model training to final evaluation on the test set, is shown in Fig. 1.

### SHAP analysis and SNP selection

Based on the outputs of the trained ML models, we computed SHAP values to interpret the breed classification results and identify influential genetic markers. We used the TreeSHAP algorithm, an exact and model-specific method for tree-based ensembles, which accounts for complex feature interactions while remaining computationally efficient.

For a given SNP ${{x}}_{i}$, the SHAP value ${{\varnothing}}_{i}$ is defined as in Eq. (1):

1 $$ \emptyset _{i} = \sum _{{{\mathrm{S}} \subseteq \left\{ {{\mathrm{P}}\backslash {\mathrm{x}}_{{\mathrm{i}}} } \right\}}} \frac{{\left| {\mathrm{S}} \right|!\left( {\left| {\mathrm{P}} \right| - \left| {\mathrm{S}} \right| - 1} \right)!}}{{\left| {\mathrm{P}} \right|!}}\left\{ {{\mathrm{f}}\left( {{\mathrm{x}}_{{{\mathrm{S}} \cup \left\{ {\mathrm{i}} \right\}}} } \right) - {\mathrm{f}}\left( {{\mathrm{x}}_{{\mathrm{s}}} } \right)} \right\} $$

where ${\mathrm{P}}$ is the complete set of SNPs, $\mathrm{S}$ is a subset excluding ${\mathrm{x}}_{i}$, ${\mathrm{f}}\left( {{\mathrm{x}}_{{{\mathrm{S}} \cup \left\{ {\mathrm{i}} \right\}}} } \right)$ is the model output when ${\mathrm{x}}_{i}$ is included, and ${\mathrm{f}}\left( {{\mathrm{x}}_{{\mathrm{s}}} } \right)$ is the output when it is excluded. The factorial weighting term $\frac{{\left| {\mathrm{S}} \right|!\left( {\left| {\mathrm{P}} \right| - \left| {\mathrm{S}} \right| - 1} \right)!}}{{\left| {\mathrm{P}} \right|!}}$ accounts for the relative likelihood of each subset size. TreeSHAP leverages the tree structure to compute these values in polynomial time, enabling analysis of large-scale genomic datasets.

Global SNP importance was quantified as the mean absolute SHAP value across all samples (*n*), defined as in in Eq. (2):

2 $$\left| {{\mathrm{mean}}\:{\mathrm{SHAP}}} \right|_{{\mathrm{i}}} = \frac{1}{{\mathrm{n}}}\sum\limits_{{{\mathrm{k}} = 1}}^{{\mathrm{n}}} | \emptyset _{i}^{{\left( {\mathrm{k}} \right)}} {\mid }$$

representing the average magnitude of each SNP’s contribution to the prediction, irrespective of direction.

SHAP values were computed on the training-validation set for each of the six repetitions and then averaged across repetitions. For each SNP, the mean SHAP value was calculated across iterations, and SNPs were ranked in descending order based on the absolute mean SHAP values to define the top 500 informative SNPs. The breed-specific relevance of these SNPs was statistically confirmed using Kruskal–Wallis tests (*p* < 0.001). Finally, two SNP panels were defined: (1) the top 500 SNPs selected based on SHAP values for LightGBM and (2) all SNPs with non-zero coefficients identified by the glmnet model. Each SNP panel was evaluated using the same individuals and identical data partitions as defined in the Data Partitioning section. For each panel, model training and hyperparameter optimization were conducted using a training–validation procedure with five-fold cross-validation and BO, followed by evaluation on the independent test sets (Fig. 1).

### SHAP–F_ST_ comparison

To validate our SHAP-based SNP importance approach, we calculated SNP-wise F_ST_ values using the Weir and Cockerham method [5]. SHAP importance values were then compared with F_ST_ estimates using Spearman’s rank correlation to evaluate the degree of overlap between SHAP-derived and F_ST_-identified informative SNPs. Correlations were computed for different SNP sets, including the top 50 and 100 SNPs from each method, with details provided in Additional file 2 Table S2. This analysis was performed as an exploratory validation of the SHAP method.

### Biological annotation

To investigate the potential biological functions of the identified SNPs, gene annotation was performed using the biomaRt package [22] in the R environment (version 4.5.1). Queries were made to the Ensembl database (release 112) through the *btaurus_gene_ensembl* dataset, based on the ARS-UCD1.2 bovine genome assembly. For each SNP, a genomic window surrounding the SNP position (50 kb) was scanned to identify all annotated genes. For each gene, we retrieved its Ensembl ID, official gene symbol, and functional description. This approach allows SNPs identified by the predictive model to be linked to candidate genes for subsequent biological interpretation.

## Results

Table 2 summarizes the test accuracy of the optimized GLMNET, RF, and LightGBM models, each tested across six random seeds to evaluate stability and robustness. The GLMNET model achieved an average accuracy of 0.817, while the RF model performed slightly better with an average of 0.837.

The LightGBM model further improved performance, with accuracies ranging from 0.835 (Seed 3) to 0.849 (Seed 4) and an average of 0.842, showing that the model is stable across different random initializations.

To examine the effect of marker selection, two reduced SNP panels were also tested. Using the LightGBM-SHAP framework, the top 500 informative SNPs reached an average accuracy of 0.880, showing that selecting important SNPs can improve classification. Similarly, the non-zero SNPs identified by the GLMNET model achieved the highest average accuracy (0.857), indicating that informative SNP pruning can further enhance predictive performance. A detailed confusion matrix for the reduced SNP panels, selected based on SHAP value and then analyzed using the LightGBM model, is provided in Additional file 3 Table S3.

Overall, these results show that LightGBM provides a reliable baseline for breed classification and that using feature-importance information either through SHAP or GLMNET can improve accuracy.

**Table 2:** Test accuracies of GLMNET, RF, and LightGBM

Partition	Accuracy				
	GLMnet	RF	LightGBM	LightGBM Top 500 informative SNPs (SHAP)	LightGBM All non-zero SNPs (GLMnet)
1	0.819	0.843	0.845	0.881	0.863
2	0.832	0.829	0.837	0.878	0.858
3	0.808	0.829	0.835	0.886	0.849
4	0.813	0.846	0.849	0.881	0.852
5	0.816	0.835	0.844	0.875	0.856
6	0.815	0.842	0.843	0.884	0.860
Average	0.817	0.837	0.842	0.880	0.857

Test accuracies were evaluated on independent test sets across six random seeds

### SHAP analysis reveals key breed-specific SNPs

**Fig. 2:**
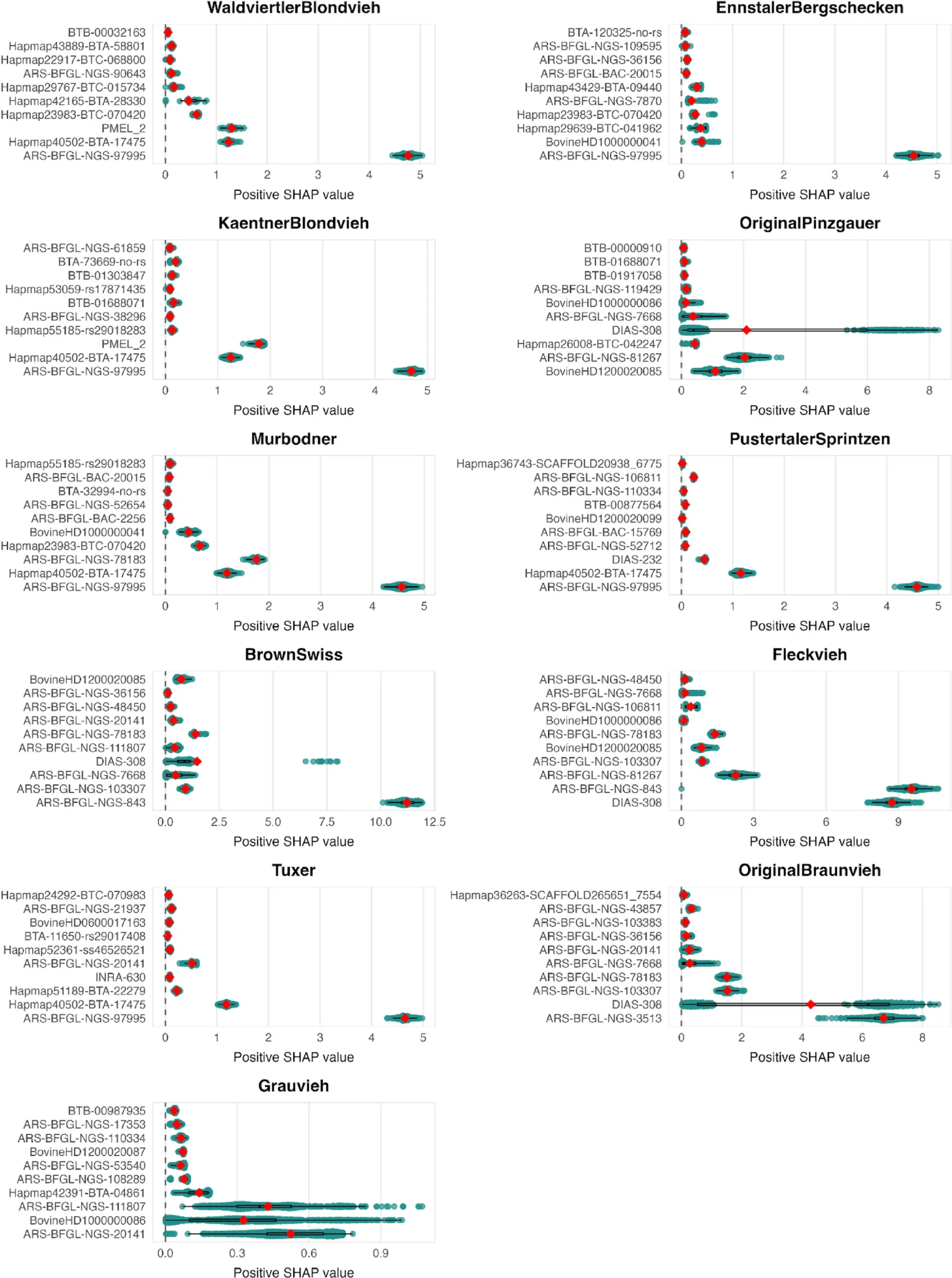
Top Positive-impact SNPs per Cattle Breed. Beeswarm plots show the distribution of positive SHAP values for the most influential SNPs in each of the eleven Austrian cattle breeds. Green dots represent individual SHAP values across six model seeds, black boxes indicate interquartile ranges, and red diamonds show mean values

**Fig. 3:**
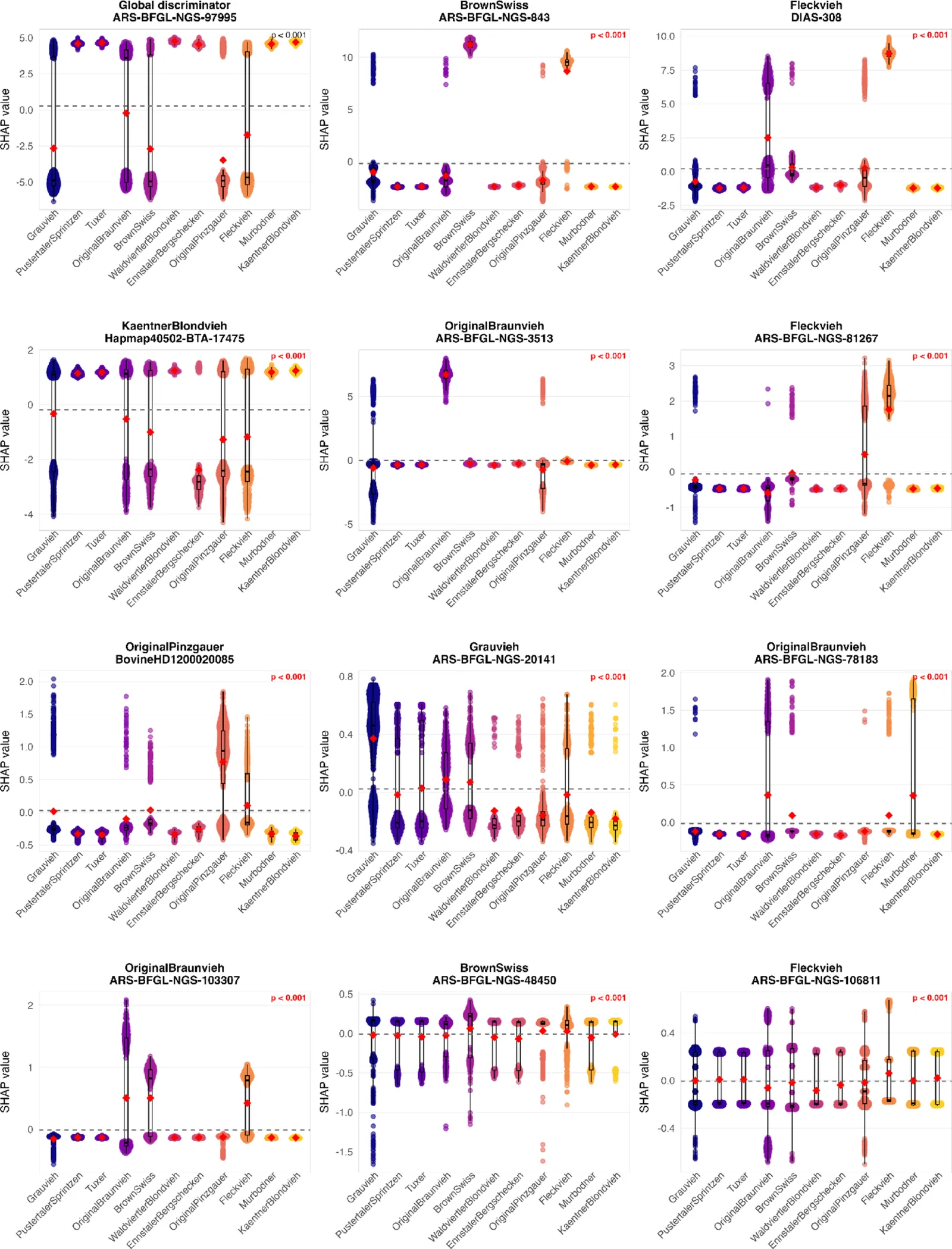
Beeswarm plot showing SHAP values for 12 key SNPs. The beeswarm plot shows the distribution of SHAP values for the 12 most influential SNPs across the eleven Austrian cattle breeds. Each dot represents an individual SHAP value from a single animal across six independent model seeds, with dot colours indicating breed origin as shown in the legend. Positive SHAP values indicate that the SNP increases the predicted probability of assignment to a given breed, whereas negative values decrease it

Our SHAP-based analysis identified a comprehensive set of informative SNPs for breed discrimination, presented through two complementary perspectives. Figure 2 lists the top ten SNPs with the highest positive SHAP values for each breed, highlighting breed-specific influential markers such as ARS-BFGL-NGS-843 for Brown Swiss and ARS-BFGL-NGS-3513 for Original Braunvieh, while also revealing SNPs shared across multiple breeds like ARS-BFGL-NGS-97995. Figure 3 provides a detailed view of the effect distribution for twelve key SNPs across all breeds, revealing their specific discriminatory roles. For instance, the beeswarm plot for ARS-BFGL-NGS-97995 demonstrates its broad importance as a global discriminator, whereas SNPs like DIAS-308 and Hapmap40502-BTA-17,475 show strong, specific effects for Fleckvieh and the Blondvieh populations, respectively, all with extreme statistical significance (*p* < 0.001). Collectively, these findings pinpoint a robust set of SNPs that define the genetic architecture of these breeds. We have selected these highly informative markers for subsequent investigations, including the analysis of candidate genes in their proximity.

### Breed-informative SNPs and nearby candidate genes

We investigated 12 key discriminative SNPs identified by SHAP (Fig. 3) to explore the genetic basis of breed differentiation. The top-ranking SNPs were annotated by obtaining their genomic coordinates and chromosome positions (Table 3), providing a basis for identifying nearby candidate genes. This analysis identified several informative SNPs that differentiate cattle breeds based on their genomic profiles. The marker ARS-BFGL-NGS-97995, located on chromosome 3 near *OR6K4*, was the most influential in stratifying overall breed groups and consistently showed high discriminatory power across multiple breeds, especially WaldviertlerBlondvieh, Tuxer, and Murbodner. In Grauvieh cattle, the SNP ARS-BFGL-NGS-20141 was identified as a breed-informative marker located near *VDAC1*. It was among the top variants that distinguished Grauvieh from other breeds. In Kaentner Blondvieh, Hapmap40502-BTA-17,475 was identified as an informative marker located close to *OR10C1*, suggesting it may play a role in breed differentiation. In Original Braunvieh, ARS-BFGL-NGS-3513 was detected as an important breed-informative SNP near *DCT* and showed clear ability to distinguish this breed from others. In Fleckvieh, DIAS-308 was identified as an informative marker near *SMIM18* and was also among the top variants separating this breed from the others. Overall, these SNPs are distributed across different chromosomes and capture genomic differences among the studied breeds. Their location near biologically relevant genes supports further functional investigation.

**Table 3:** Genomic coordinates and gene annotations of top-ranking SNPs identified by the predictive model

SNP	CHR	POS	Genes symbol
ARS-BFGL-NGS-103307	1	118,048,415	–
ARS-BFGL-NGS-97995	3	10,941,192	*OR6K4*
ARS-BFGL-NGS-20141	7	45,691,037	*VDAC1*
ARS-BFGL-NGS-78183	9	14,390,037	–
BovineHD1200020085	12	72,174,274	–
ARS-BFGL-NGS-3513	12	68,998,339	*DCT*
ARS-BFGL-NGS-843	14	74,059,949	–
ARS-BFGL-NGS-48450	14	74,037,636	–
ARS-BFGL-NGS-81267	23	10,570,333	*RPS4Y1*
Hapmap40502-BTA-17475	23	29,137,447	*OR10C1*
ARS-BFGL-NGS-106811	23	10,548,475	–
DIAS-308	27	26,738,014	*SMIM18*

### Overlap between breeds

**Fig. 4:**
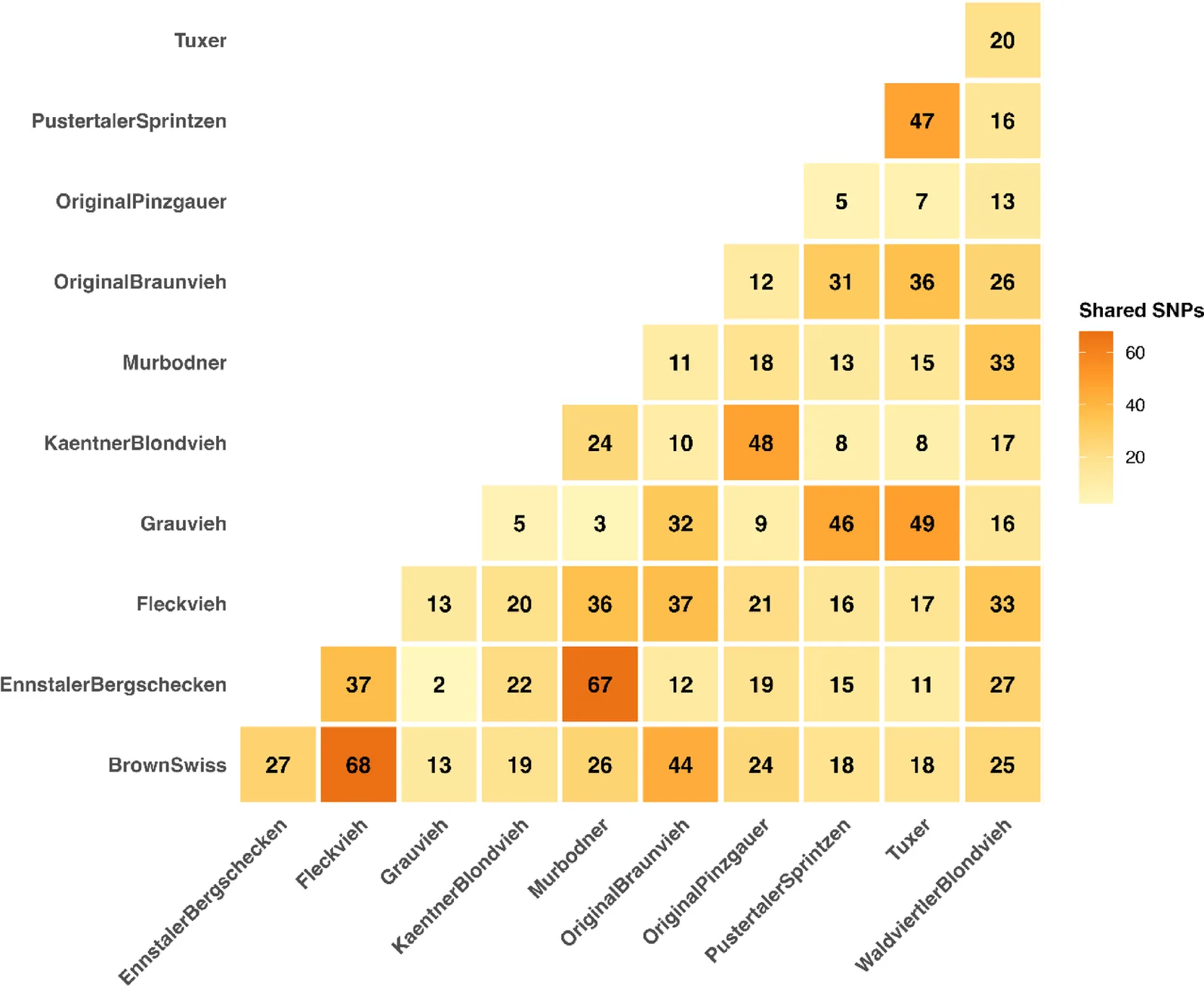
Pairwise overlap of top SHAP-ranked SNPs among breeds. Heatmap showing the number of shared SNPs among the top 100 positive-impact SNPs for each of eleven Austrian cattle breeds

Similarities in predictive genomic signals among breeds were evaluated by quantifying pairwise overlap of informative markers. For each breed, the top 100 SNPs with the highest positive SHAP values were selected, and the number of shared SNPs was calculated for each breed pair. The highest number of shared SNPs were observed between Brown swiss and Fleckvieh (68), Murbodner and Ennstaler Bergschecken (67), and Tuxer and Grauvieh (49). These shared high-ranking SNPs reflect common model-derived predictive signals among breeds (Fig. 4).

## Discussion

### A novel framework and its applications for genomic breed assignment

Based on our knowledge, no prior study has applied SHAP in conjunction with genome-wide SNP data for the classification of endangered cattle breeds. In this study, we introduced an approach by combining the power of the LightGBM model with SHAP-based interpretability to both classify and biologically interpret SNP markers across eleven endangered cattle breeds. In many existing studies, preprocessing pipelines are applied before classification. These pipelines typically include dimensionality reduction techniques such as PCA or feature aggregation methods like F_ST_ to identify a reduced set of informative SNPs [4, 13]. The selected SNPs are then used as input for a separate classification algorithm, such as RF [8] or deep learning-based models. For example, Manzoori et al. [23] applied deep neural networks together with Garson and Olden methods to select discriminant SNP panels prior to breed classification. In contrast, our approach eliminates multi-stage preprocessing steps by performing classification directly on raw SNP data, thereby preserving the full genomic information that may be reduced by linear dimensionality reduction methods. Subsequently, SHAP-based feature importance can be used to identify informative SNP subsets. Our results show that these reduced SNP panels derived from the model can maintain or even improve classification accuracy, indicating that model-based feature selection can capture the most informative signals while reducing dimensionality. Moreover, integrating SHAP values enables us to quantify the individual contribution of each SNP to the classification outcome. The benefits of using adapted SHAP have been demonstrated in various domains, including clinical data, where it has proven valuable in interpreting machine learning models and uncovering relationships between features and outcomes [24]. A recent study also showed the use of SHAP on genomic data for cancer, improving model interpretability and aiding in the identification of key biomarkers [25].

In addition, Lundberg et al. [16] showed that tree-based models, particularly gradient-boosted trees, often perform strongly on tabular datasets and can provide improved interpretability through SHAP-based explanations. Their work introduced tools that enable both local (individual-level) and global (population-level) insights into model behavior, helping to reveal non-linear feature interactions and identify potentially unstable predictors over time. In our context, SHAP explanations improve the transparency of breed classification models by highlighting the most informative SNPs.

### Biological insights and implications

Analysis of the top-ranking SNPs and nearby candidate genes demonstrated that several markers are positioned close to genes associated with key traits, including olfaction, production, fertility and coat color. In the following, we provide a detailed explanation of these associations. For example, the most important marker for breed classification (Table 3), ARS-BFGL-NGS-97995, was found on chromosome 3 near *OR6K4*. Genes in the olfactory receptor (*OR*) family, like *OR6K4*, are important for the sense of smell, which helps mammals find food and avoid danger. Studies in vertebrates show that the number of *OR* genes vary a lot between species, suggesting that the sense of smell has been shaped by natural selection [26]. *OR* genes can also affect production traits. For example, differences in *OR* genes can influence appetite, which changes feed intake, weight gain, and body composition in livestock [27]. In Grauvieh cattle, the SNP ARS-BFGL-NGS-20141 was identified as a breed-informative marker located near *VDAC1*, a gene associated with mitochondrial function and metabolic regulation [28]. In Original Braunvieh cattle, the SNP ARS-BFGL-NGS-3513 was located near *DCT* (dopachrome tautomerase), a gene known to affect coat color [29], highlighting a candidate locus that may contribute to phenotypic variation in this breed.

Our study presents a transparent and interpretable pipeline for breed classification. The combination of LightGBM with SHAP provides high predictive accuracy and interpretability at the level of individual SNPs. To evaluate SHAP, we applied the F_ST_ method and compared SNP-wise F_ST_ values with SHAP importance values using Spearman’s rank correlation. Among the top 50 SNPs identified by each method, 19 SNPs overlapped, corresponding to a correlation of 0.829, indicating that SHAP reliably highlights informative SNPs. Correlation analyses for different SNP sets showed that the top 50 SHAP SNPs had a Spearman correlation of 0.571, and the top 50 F_ST_ SNPs had a correlation of 0.598. (Additional file 2 Table S2). The developed framework has practical potential for supporting the conservation of endangered Austrian cattle breeds. By enabling accurate breed assignment based on genome-wide SNP data or reduced informative SNP panels, the model could be used as a decision-support tool in conservation and breeding programs. In particular, it may help verify breed identity and support the selection of animals for conservation herds.

### Limitations and future directions

Our framework can be updated as new data become available, or applied to other populations, including commercial breeds. However, some general limitations should be considered. The results are based on the available dataset and should be interpreted within this context. In addition, the analysis focused on individuals with confirmed breed information, which may not fully reflect practical breeding situations where crossbred or admixed animals are common. Future studies could benefit from using more diverse datasets that include a wider range of genetic backgrounds. The inclusion of admixed or crossbred individuals may improve the relevance of the approach for real-world applications. Furthermore, exploring the biological function of informative SNPs may provide additional insight into genetic variation among breeds.

## Conclusion

In this study, we showed that combining LightGBM with SHAP provides a reliable and interpretable framework for cattle breed classification using genome-wide SNP data. The model achieved classification accuracy (0.842 for LightGBM and 0.837 for RF) across multiple runs, effectively distinguishing among eleven endangered Austrian cattle breeds. Importantly, SHAP-based analysis allowed us to identify the top-ranking SNPs, several of which are located near genes associated with key traits, including olfaction, production, fertility, and coat color.

The identified SNPs can be directly used in genomic-guided conservation programs. By targeting markers specific to certain breeds or shared among related groups, conservationists can manage genetic diversity, and design breeding strategies that preserve breed identity.

Beyond Austrian cattle, this framework can be applied to other endangered livestock populations with available genomic data. The combination of high predictive accuracy and interpretability makes LightGBM + SHAP a useful tool in conservation genomics, providing practical insights for sustainable management of animal genetic resources.

## Supplementary Information

Below is the link to the electronic supplementary material.

Supplementary Material 1

Supplementary Material 2

Supplementary Material 3

## Data Availability

The datasets analysed during the current study were provided under a confidentiality agreement and are not publicly available. However, the data may be made available by the corresponding author on reasonable request and with permission of the data-providing company.
