# Correction: Interpretable machine learning for cattle breed classification and SNP prioritization

**DOI:** 10.1186/s12711-026-01083-4

**Published:** 2026-08-24

**Authors:** Farzad Atrian-Afiani, Gábor Mészáros, Johann Sölkner, Patrik Waldmann

**Affiliations:** 1 Universität für Bodenkultur Wien, Vienna, Austria; 2 Research Unit of Mathematical Sciences, University of Oulu, P.O. Box 8000, 90014 Oulu, Finland

**Correction: Genet Sel Evol 58, 37 (2026)**

10.1186/s12711-026-01056-7

Following publication of the original article [1], the authors identified a typesetting error in the author name of Johann Sölkner.

The incorrect author name is: Johann Sölker.

The correct author name is: Johann Sölkner.

The author group has been updated above and the original article [1] has been corrected.
